# Father involvement and emotion regulation during early childhood: a systematic review

**DOI:** 10.1186/s40359-024-02182-x

**Published:** 2024-11-19

**Authors:** Nilo Puglisi, Valentine Rattaz, Nicolas Favez, Hervé Tissot

**Affiliations:** 1 Faculty of Psychology and Educational Sciences, University of Geneva, Geneva, Switzerland; 2 Center for Family Studies, Department of Psychiatry, Lausanne University Hospital and University of Lausanne, Prilly, Switzerland

**Keywords:** Father involvement, Emotion regulation, Early childhood, Systematic review, Father-child relationship

## Abstract

**Background:**

Father involvement, defined in terms of both the quantity and quality of ways in which fathers may be involved, affects the child’s development. How specifically father involvement links to emotion regulation during early childhood (0–5 years) is, however, less clear.

**Methods:**

This literature review synthesizes research on the links between father involvement and emotion regulation during early childhood, as well as the measurement methods used to assess them. Ten relevant studies were identified via four databases (up to August 2023).

**Results:**

Results showed no significant direct links, but significant links appeared between high father involvement and more adaptive emotion regulation when moderated by variables related to the assessment of father involvement and emotion regulation, as well as the characteristics of the father and the child.

**Conclusions:**

Future research should continue to use observational measures of father behaviors and child emotion regulation, increase the use of physiological measures of emotion regulation, and consider the influence of maternal and family variables.

**Supplementary Information:**

The online version contains supplementary material available at 10.1186/s40359-024-02182-x.

## Background

### Introduction

Over the last 40 years of research, father involvement has emerged as a determining factor in children’s social and emotional development. Defined in terms of both the quantity and quality of ways in which fathers may be involved, father involvement is acknowledged as a factor that influences the development of emotion regulation (ER) capacities in children, which are central processes in their early socio-affective development [1–3]. ER can be defined as “the extrinsic and intrinsic processes responsible for monitoring, evaluating, and modifying emotional reactions, especially their intensive and temporal features, to accomplish one’s goals” [4, pp. 27–28]. The development of ER is a central process, as the competence of a child to regulate emotions may prevent several mental health disorders and sustain the development of positive skills (e.g., prosocial behaviors and the ability to cope with stress) [5–9]. Some studies in the field have, however, yielded contrasting results regarding the nature of the contribution of father involvement to ER development in children. To date, no review has been dedicated to synthesizing findings on this topic regarding children during early childhood (0–5 years), despite ER being of paramount importance during the first years of life. The aim of this review was thus to fill this gap by synthesizing the results of previous studies and the measurement methods that have been used.

### Father involvement and early ER in children

Despite growing recognition of the role of paternal involvement in children’s development, there are fewer studies on its influence compared with that of maternal involvement, especially in early childhood [1, 2]. Several factors may explain this difference. First, for several decades (until 1970–1980), studies on parenting, especially in the early childhood period, focused only on mothers, primarily because most households were traditionally organized according to specialized roles, with mothers taking care of the child(ren) and fathers being the breadwinners. Since the 1970s, sociocultural changes in the conception of fatherhood and family organization have led to a general increase in the involvement of fathers during early childhood in Western countries. An imbalance between women and men in family life still exists, however, and mothers, whether working or not, still assume the role of primary caregiver for children in most families [10]. Indeed, there is far more variation in father involvement than there is in mother involvement toward children. This imbalance is also spurred by family policies in many Western countries, where mothers are encouraged to take (or receive) maternity leave, whereas paternity leave remains marginal or even nonexistent in some countries [11]. Although imbalance persists, the fact remains that father involvement with young children has increased steadily over the past 50 years. As a consequence, scholars have started to conduct more studies about father involvement and its influence on child development.

Research on fatherhood has shown that fathers play a unique role in children’s development, as they exhibit distinct parenting behaviors compared with those of mothers. For example, fathers engage more in physical play, encourage children to take risks, are more likely to help children deal with scary situations, and elicit higher emotional arousal in children during interactions [12, 13]. The unique parenting behaviors displayed by fathers have suggested that their involvement can have a unique influence on the cognitive, social, and emotional development of their children. This suggestion has been highlighted by previous studies, whose results have been synthesized in several reviews, although they did not specifically focus on ER [14–19]. An exception is a recent literature review that specifically emphasized the role of fathers in the child’s ER development during the first 18 years of life, revealing that “a good modelling of ER by fathers, supportive emotion-related parenting practices, and a positive father–child emotional climate were associated with higher ER skills in children” [20, pp. 35]. However, this review did not specifically focus on father involvement during the early childhood period (0–5 years), a pivotal period for socioemotional development during which the majority of self-regulatory abilities are built and parents’ involvement is crucial in shaping them [21, 22]. During the early childhood period, children typically move from reactive, coregulated emotions during interactions with adults (primarily parents in the early years) to more advanced forms of emotional, cognitive, and behavioral self-regulation in different life environments (e.g., family, and school) [23, 24]. The progression toward more advanced forms of self-regulation is a continuous process that can significantly be influenced by challenges experienced in the early years (e.g., the transition from home to formal schooling) [6–8]. Father involvement may increase or decrease the quality of the children’s ability to self-regulate. In summary, the early years of life are crucial for children’s socioemotional development as they become progressively able to self-regulate. Within this period, the active involvement of fathers plays a crucial role in the ongoing process of developing ER skills, as children depend on others, especially the parents, to learn how to self-regulate emotions.

### Models and measures of father involvement and child’s ER

The fields of research on father involvement and ER are marked by methodological, conceptual, and theoretical heterogeneity. This heterogeneity within both fields poses challenges in forming a clear picture about which aspects of father involvement relate to those of ER. Regarding the field of research on father involvement, a first observation is that there is no consensus on a model conceptualizing father involvement and that many conceptual and theoretical models have been formulated and serve as conceptual bases for operationalizing father involvement in research–with psychosocial models of father involvement being the most frequent (e.g., Lamb and colleagues’ [25] tripartite model; Paquette’s [26] theory of the father–child activation relationship; Pleck’s [27] multidimensional model of father involvement; Cabrera and colleagues’ [28] heuristic model; Palkovitz & Hull’s [29] resource theory of fathering; Volling & Cabrera’s [30] developmental ecological systems framework). Although these models partially overlap each other, each may contain a different set of central dimensions (e.g., direct engagement, accessibility, warmth, control, responsibility, presence/absence of fathers, frequency of caregiving, physical and cognitively stimulating activities, indirect care), which may make it difficult to synthesize the results of studies in the field [31]. A second observation is that there is no clear consensus on the naming of phenomena related to father involvement. For example, interchangeable terms, such as “father involvement,” “father engagement,” and “fathering,” have been used to refer to “father involvement” [32]. Nonetheless, there is a relative consensus that fathers can be involved in different ways (such as directly interacting with the child, overseeing activities, and meeting their needs) and that the concept of father involvement can refer either to the quantity (time spent with the child) or quality (the nature of paternal behavior during interactions with the child) [27, 31, 33] of the involvement of the father with the child. However, this consensus is less generalized when it comes to determining how to measure the quantity and quality of father involvement. Indeed, a third observation is that there is no clear consensus on the “optimal” method for assessing father involvement. This lack of consensus on assessment methodologies poses a significant challenge in synthesizing the results of the field. This is because different sets of surveys, behavioral observations, and interviews have been used in the research to assess the quantity and quality of father involvement. Survey-based research has mainly focused on the quantity of father involvement in order to assess, for example, the number of tangibles: concrete activities that fathers perform for or with their children and the amount of time (e.g., hours per day, days, or times per week) spent together [34, 35]. Through surveys or interviews, studies have also investigated the quantity of continued presence of fathers in the household, in the child’s life, or in providing economic support to the mother [36, 37]. When researchers could not easily recruit fathers, they often used mothers to obtain information on father involvement in terms of the quantity and continuous presence of the father, which potentially leads to biased data [38, 39]. Conversely, observational measures have often been used to investigate the quality of father involvement as, for example, the quality of the father-child relationship or parenting style [40, 41]. Studies on the quality of father involvement have also resorted to surveys, observations, or interviews to assess overall indicators of father involvement. These overall indicators typically encompass information on various domains of father involvement such as care, hygiene, nurture, discipline, learning, and play with the child [42–45].

Similar to the field of research on father involvement, research on ER in young children has used several measures for assessing ER, leading to debates on how each measure may be the “gold standard” to assess children’s ER [46–48]. Previous reviews have highlighted three main methods to assess ER in young children, to which the present paper will refer: informant report (parent, teacher, or peer), naturalistic or laboratory observation, and physiological-biological indicators. As self-reports are not an appropriate method in early childhood because of the limited verbal capacities of infants and toddlers, researchers have frequently resorted to informant reports (mostly from parents). Informant reports have the advantage of being quick to administer and easy to analyze; however, they are subject to various biases, including extreme responses and social desirability influences [49]. Existing questionnaires (for a review, see [50]) investigate different aspects of children’s ER, such as the degree of emotion dysregulation, the use of regulatory strategies, or overall ER scores calculated from one or more subscales of a child’s development questionnaire (for a review of different aspects of ER assessed by self- and informant reports, see [48]). Naturalistic or laboratory observation measures have frequently been used to assess children’s ER during early childhood. They often involve situations that elicit emotions of frustration, anger, or fear in the child (e.g., the mother disappears for a few minutes and the child remains alone in the room [51]). The behaviors that the child displays during these situations can be coded by the experimenter as ER strategies, distress regulation, and recovery from a stressful situation. In non-stressful situations, (e.g., free play [52]), designed to elicit no specific response from the child, the child’s behaviors can be assessed as strategies of emotion management [53]. Although observational methods allow researchers to objectively measure ER in children, their administration and analysis are time-consuming. Physiological-biological indicators are also used to assess physiological or neural processes involved in ER [54, 55]. These indicators (e.g., heart rate variability and cortisol [56, 57] provide an objective measure of the processes involved in ER, which are crucial in the modulation of ER behavior. However, each indicator allows for measuring one aspect of ER processes, and so it is recommended to use them in combination with other ER measures [58]. The heterogeneity of measures for assessing ER makes it necessary to summarize which have been used in relation to father involvement so that the links between the two constructs can be better identified, gaps highlighted, and future research guided.

Taking these considerations about father involvement and ER into account, in the present paper, we aimed to (i) synthesize the literature on the links between father involvement and ER during early childhood, (ii) describe the methods used to study the link between father involvement and ER in children’s first 5 years, and (iii) identify and discuss gaps in the literature and provide directions for future research on the link between father involvement and children’s ER in early childhood.

## Method

**Fig. 1:**
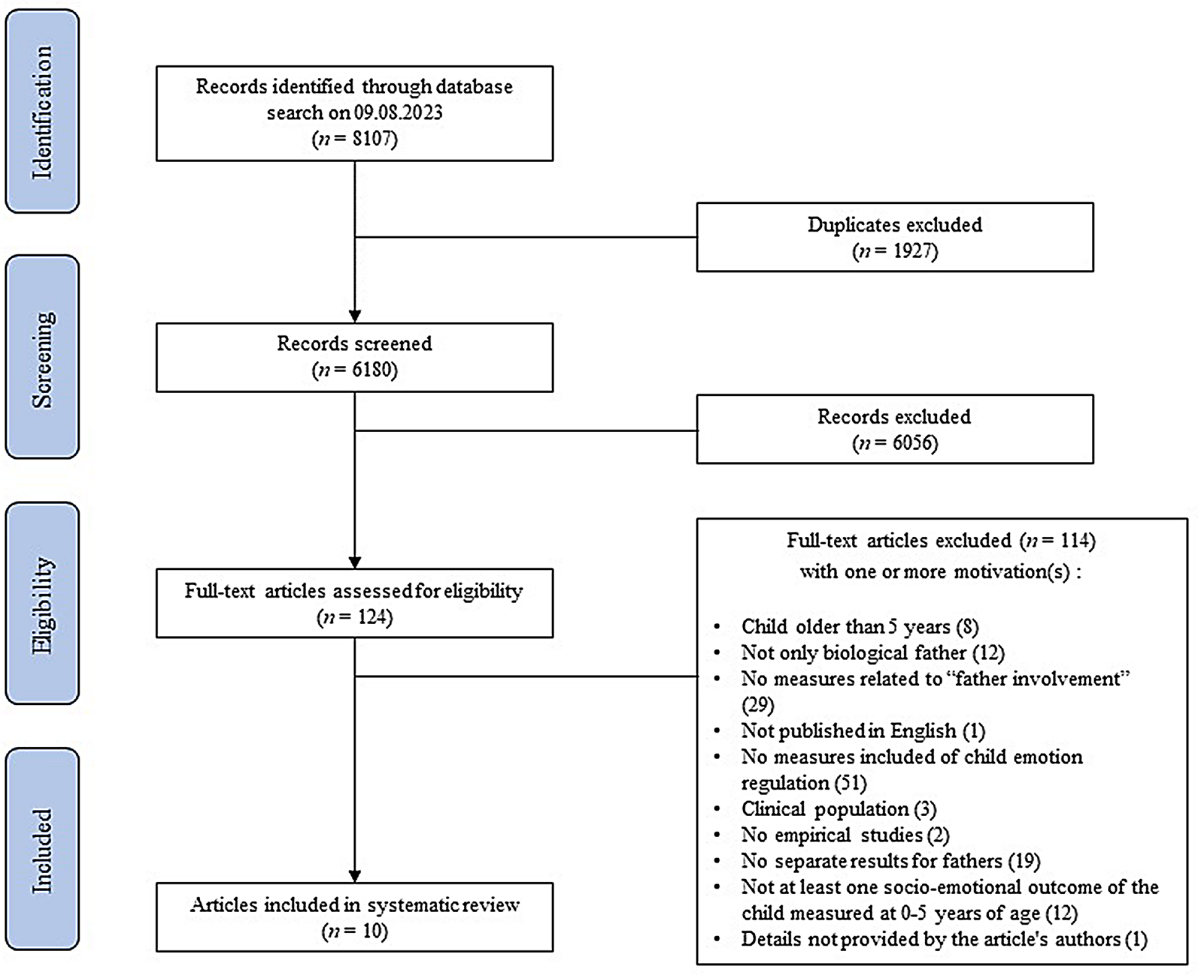
PRISMA Flow Diagram

The systematic review protocol was developed by following the Preferred Reporting Items for Systematic reviews and Meta-Analyses (PRISMA; see Fig. 1) recommendations [59].

A research algorithm was created with terms related to father involvement (e.g., “father accessibility,” “paternal involvement”) and ER (e.g., “socioemotional development,” “affect regulation,” “psychophysiology”). Search algorithms are available online as additional file 1. Four international web databases were searched (PubMed, PsycNET, EMBASE, and Web of Science) by the first author on August 9, 2023. The articles were selected by the first and second authors according to the following criteria: (I) studies including men presented as involved paternal figures, despite marital status or biological relation; (II) studies involving 0- to 5-year-old children; (III) studies involving no fathers or children with a diagnosis that affects ER; (IV) studies with at least one measures related to the quantity and quality of “father involvement”, as referred to in psychosocial models of father involvement; (V) parenting studies that distinguished results for fathers from those for other caregivers (e.g., mother); (VI) studies reporting at least one measure of child ER collected between 0 and 5 years of age; (VII) studies published in a peer-reviewed journal; (VIII) studies published in English; (IX) only empirical studies; and (X) no guidelines, syntheses, systematic or non-systematic reviews, meta-analyses, perspective articles, or theoretical or conceptual models.

Studies identified in database searches were exported to EndNote 20.1. Two reviewers (first and second authors) independently evaluated titles and abstracts, and then full texts. Consensus meetings were held at each stage to determine progression to the next stage, and the fourth author resolved discrepancies. Reasons for exclusion were documented in a spreadsheet and disagreements were resolved by consensus. A total of 8107 studies were found from the extended search. After duplicate articles were removed (*n* = 1927), 6056 non-relevant articles were excluded based on the title and abstract. The remaining 124 articles were screened based on the full text, and 114 studies were excluded according to the inclusion/exclusion criteria. Finally, 10 articles were included in the systematic review, with data derived from 10 studies. The extended data details from the included articles are available in Tables 1 and 2 and the additional file 2.

## Results

We organized the results by grouping the studies according to the two categories in which father involvement can be defined, namely, quantity and quality of father involvement. For each of the two categories of father involvement, we present the studies that have measured ER as informant reports, naturalistic or laboratory observation, and physiological-biological indicators.

### Population

Several studies included in this review reported partial or incomplete information about the characteristics of their samples, thus limiting an accurate summary of available characteristics (see Table 1 and additional file 2).

**Table 1:** Characteristic of the sample

Characteristic of the sample	*n*	Study
*Infants/toddlers’ category of age*		
Infant (0–12 months)	3	63, 69, 71
Toddler (13–35 months)	3	60, 62, 70
Pre-schoolers (3–5 years)	3	68, 72, 75
Mixed ages	1	61
*Country of father’s origins*		
USA	9	61–63, 68–72, 75
Italy	1	60
*Fathers’ Race/Ethnic background*		
Majority European American	6	61, 63, 68–71
Mixed	3	62, 72, 75
Not applicable/available (N/A)	1	60
*Fathers’ education*		
Majority university	5	60, 61, 68, 70, 71,
Mixed (i.e., no formal education, primary, secondary, university)	4	63, 69, 72, 75,
N/A	1	62
*Fathers’ socioeconomic status (SES)*		
Middle	4	68, 70, 71, 75
Low	1	62
Mixed SES (i.e., a mix of lower, middle, and upper SES)	3	61, 63, 69
N/A	2	60, 72
*Fathers’ category of age*		
Mixed ages (i.e., range 14–63 years)	5	61–63, 68, 69
Young adults (18–35 years)	3	60, 70, 71
Age data not indicated	2	72, 75
*Mother and father living together*		
Yes	6	61, 63, 68–71
Mix (i.e., parents living and not living together)	3	62, 72, 75
*Fathers’ marital status*		
Married	1	61
Married or cohabiting	2	70, 71
Majority married	3	62, 72, 75
Majority married or cohabiting	2	68, 69
N/A	2	60, 63
*Parents’ sexual orientation*		
Heterosexual	3	60, 69, 70
N/A	7	61–63, 68, 71, 72, 75

All studies were conducted in the United States except for one (Italy). Of the 10 studies included, seven were longitudinal, and three were cross-sectional. While cross-sectional studies offer advantages such as cost-effectiveness, time efficiency, and simplicity, their presence within the limited number of included studies may hinder a deeper understanding of the causal relationships between the target variables in early childhood development. Nevertheless, they still offer valuable insights that can guide future research (see Discussion). Fathers’ education was diverse in most studies, ranging from no formal education to university education. Although most studies did not consider or provide more details on fathers’ socioeconomic status, the available information indicates that a majority were middle-class fathers, except for three studies with a mix of lower-, middle-, and upper-class fathers, and one study with low-income fathers. In seven of the 10 included studies, fathers lived with the mother and their child. Children’s mean age ranged from 3 to 42.36 months. Of the 10 studies included, three focused on infants (0–12 months), three on toddlers (13–35 months), and three on pre-schoolers (3–5 years), whereas one contained mixed ages. Fathers’ mean age ranged from 25.51 to 35.86 years. Most of the included studies focused on fathers of mixed ages (i.e., range 35–64 years; *n* = 6), two studies focused on young fathers (18–35 years), and two studies did not mention the age of the fathers. The living arrangements of families were reported as follows: not specified (*n* = 2), married (*n* = 1), married or cohabiting (*n* = 2), mostly married (*n* = 3), mostly married or cohabiting (*n* = 2). All studies focused on different-sex parents; of these, only three explicitly mentioned the parents’ sexual orientation, identifying them as heterosexual. Since it cannot be assumed that all different-sex parents are heterosexual, we suggest that future research specifies the sexual orientation of the parents. Including information about the parents’ sexual orientation would provide a more accurate description of the sample. It would also enable an analysis of differences related to sexual orientation, such as how challenges related to social support, cultural norms, prejudices, and parenting models affect father involvement in families with non-heterosexual parents. In six of the 10 included studies, there was a disparity in the sample between female and male infants or toddlers. Eight of the 10 included studies were published after 2019, indicating a recent trend in investigating the links between father involvement and ER during early childhood. The data collection period was approximately deduced by using available information, such as the reported study’s grant number, as none of the included studies explicitly indicated the years in which the data were collected. In two of the 10 studies, details about the data collection period were unavailable. In the remaining eight studies, data collection occurred before 1999 in one study, between 2000 and 2015 in three studies, and after 2016 in four studies. Considering the information about the publication years and data collection years in the included studies, we recommend that forthcoming research explicitly specify the years of data collection. This step could improve the interpretability of the results in the context of socio-cultural changes related to the conception of fatherhood and the roles assumed by “new” fathers.

### Methodological characteristics

#### Father involvement

Of the 10 included studies, five considered father involvement in terms of quantity, four in terms of quality, and one in terms of both quantity and quality, for a total of six studies considering the quantity and five considering the quality of father involvement. In these studies, the quantity of father involvement was reported by fathers (*n* = 4), mothers (*n* = 1), or both parents (*n* = 1). In all five studies that assessed the quality of father involvement, researchers used direct observation of father-infant interactions. Among the included studies, the quantity and quality of father involvement were measured by referring to several aspects of father involvement, in many cases considering two or more aspects simultaneously. In most of the studies targeting the quality of father involvement, the authors considered a set of the fathers’ behaviors during interaction with the child, computing global or subscale scores (e.g., the average score of the father’s positive emotions coded for each 30-s interval of interaction with the child) that indicated the quality of involvement during these interactions (*n* = 5). The remaining studies focused on the quantity of father involvement in diverse care, play, and teaching activities (*n* = 4). One study focused on father involvement as the quantity of fathering styles (e.g., fathers’ authoritative and permissive parenting), and one study investigated father involvement as the father’s continuous physical presence or absence. Of the 10 studies included, three assessed father involvement during the first 12 months of infants’ lives, three when toddlers were between 13 and 36 months old, and three when pre-schoolers were between 3 and 5 years old. In the remaining study that assessed father involvement in terms of both quantity and quality, the assessment of quantity was conducted within the infant’s first 12 months of life, whereas the assessment of quality occurred once within the initial 12 months and then again when the toddler was 24 months.

#### Emotion regulation

Most studies examined ER during naturalistic or laboratory observation (*n* = 4), and researchers assessed children’s ER during interactions with fathers (*n* = 1), during interactions with both parents (*n* = 1), or during tasks with no parents involved (*n* = 2). Of the remaining studies, three investigated ER with informant reports (father’s report, *n* = 1; mother’s report, *n* = 1; both parents’ report, *n* = 1) and three as physiological-biological indicators. Of the 10 studies included, three assessed ER during the first 12 months of infants’ lives, four when toddlers were between 13 and 36 months old, and three when pre-schoolers were between 3 and 5 years old.

### Narrative synthesis of the results

**Table 2:** Main findings from the studies

Authors	Main findings
De Stasio et al. [60]	There were no correlations between paternal bedtime and global involvement with the paternal report of a child’s emotional lability/emotion regulation. Correlations were present with the maternal report of the child’s emotional lability/emotion regulation, such that when the father involvement was low, the greater were the emotion regulation difficulties reported by mothers (*r* = − .35, *p* < .01, bedtime involvement; *r* = − .27, *p* < .05, global involvement).
Aquino et al. [61]	Children of fathers who were disengaged during an interaction with them at 8 months displayed more emotional underregulation at 24 months (*β* = 0.27, *p* < .01). More displays of paternal minimizing responses were related to greater child emotional underregulation at 24 months (β = 0.41, *p* < .001). Greater father involvement was related to greater children’s emotional underregulation at 24 months (β = 0.191, *p* < .05).
Bocknek et al. [62]	Greater consistent biological fathers’ presence correlates with greater child emotion regulation at 24 months (*r* = .06, *p* < .05) and 36 months (*r* = .07, *p* < .05), but not at 14 months. Consistent biological fathers’ presence links to toddlers’ regulatory development across toddlerhood, particularly among Caucasians as compared with African American toddlers (effect size not reported).
Planalp & Braungart-Rieker [63]	Infants lower in Surgency with a highly involved father increased Self-distraction at a faster rate (but not in Self-comforting regulatory strategy), particularly with highly involved fathers (effect size not reported).
Isaac et al. [68]	Greater fathers’ authoritarian parenting (*r* = .23, *p* < .05) and physical coercion (*r* = .26, *p* < .05) correlated with higher hair cortisol concentration. Fathers’ authoritative and permissive parenting and fathers’ non-reasoning/punitive did not correlate with children’s physiological stress.
Richter & Lickenbrock [69]	At 4 months, infant cardiac physiology (i.e., RSA) correlates with father involvement in play (*r* = .22, *p* < .05) and does not correlate with father involvement in care. At 8 months, infant cardiac physiology (i.e., RSA) correlates with father involvement in care (*r* = .36, *p* < .01) and does not correlate with father involvement in play. Infants with highly involved fathers in care have higher baseline RSA (*β* = 0.34, *p* = .001), typically associated with better emotion regulation. Fathers’ play is not significant.
Olofson and Schoppe-Sullivan [70]	The father’s parenting behaviors are not associated with the mother’s infant-toddler dysregulation.
Altenburger & Schoppe-Sullivan [71]	Children’s negative emotionality score does not correlate with paternal sensitivity, detachment, and positive affect. Children’s orienting and regulatory capacity score correlate with positive affect (*r* = .16, *p* < .05) and do not correlate with paternal sensitivity and detachment.
Lunkenheimer et al. [72]	Higher paternal responsiveness and expressiveness were both not related to children’s lower negative arousal with fathers. However, they were both related to children’s lower negative arousal with mothers (*r* = − .295, *p* < .01, paternal responsiveness; *r* = − .237, *p* < .05, paternal expressiveness).
Burniston et al. [75]	Greater paternal supportive emotion socialization was significantly associated with children’s higher total cortisol output (*β* = 0.31, *p* < .05).

### Quantity of father involvement

#### ER as informant reports

The only study that investigated informant-reported ER found no significant direct link between the variables, but significant links appeared that were based on the informant. Indeed, links between the greater quantity of father involvement and better child ER appeared when the mother, not the father, reported the quantity of father involvement [60]. Although no definitive conclusion can be drawn, the results of this particular study encourage investigations of the links between the quantity of father involvement and the reported ER and how the informants may reveal these links.

#### ER as a naturalistic or laboratory observation

Of the three studies that investigated observed ER, only one found significant direct links, notably between the greater quantity of father involvement and better child ER [61]. The other two studies found that significant links between the greater quantity of father involvement and better child ER appeared when other variables were considered: assessment time of ER and father’s ethnicity [62], and assessment characteristics of ER and measured aspects of the infant’s temperament [63]. In the first of the two studies, Bocknek et al. [62] found positive links between mothers’ reports of a more consistent father presence and greater child ER, as evaluated by researchers. Notably, these links were significant at 24 and 36 months, but not at 14 months. In addition, positive links were observed for fathers of Caucasian ethnicity, but not for fathers who were African-American or Hispanic. These findings highlight the cultural aspect inherent in the concept of involvement, as proposed by certain fatherhood models [64], and align with the idea that a father’s influence evolves during a child’s development [65]. Nevertheless, caution is warranted in interpreting the results of Bocknek et al.‘s [62] study due to the potential inclusion of separated couples in the sample. Specifically, half of the fathers either lived separately from or were not married to the mothers, suggesting the plausible presence of separated parents. In cases of separation or divorce, various factors could have influenced mothers’ reports of father involvement, including parental conflicts, family organization, and economic challenges—especially considering the low-income fathers observed in the study by Bocknek et al. [62]. The impact of these factors, alongside the coexistence of families with both separated and non-separated parents, might have contributed to the weakening of significant direct links between the constructs of interest across the entire study sample. Future investigations should take into account these considerations and the influence of family variables, especially in the case of separated or divorced parents. In the second of the two studies, Planalp and Braungart-Rieker [63] found that the fathers’ report of their greater involvement at home influences infant ER during infant-father interactions. However, links were moderated by the assessment of ER and the infant’s temperament. Indeed, only infants lower in surgency (a dimension of temperament) increased in self-distraction faster when father involvement was higher and slower when father involvement was lower. No links were found in the analyses that considered self-comforting as a regulatory behavior, and negative affectivity and regulation as infants’ dimensions of temperament. These results highlight the pivotal role of children’s temperament in shaping their regulation, emphasizing the need for future research to incorporate this aspect. In addition, they underscore the multidimensional nature of ER, suggesting that its links with the quantity of father involvement may vary based on the specific dimension of ER measured. Nevertheless, it is crucial to acknowledge that this was the only study that exclusively depended on the father’s reports of the infant’s temperament, omitting input from the mother, for example. Moreover, regulatory strategies were observed during the Still Face Paradigm [66], a scenario designed to specifically elicit frustration in infants, which might potentially constrain the generalizability of the findings to other emotions, such as fear. To address these considerations, future studies should include multiple measures of infant temperament (e.g., from multiple informants) and examine determinants of regulatory behaviors in various emotion-eliciting contexts.

When we compared the three included studies, we identified some variables that differed across studies and might have influenced the observed differences in their results: timing of data collection, sample size, assessment time of ER and father involvement, assessment characteristics of ER and father involvement, informant of father involvement, father’s socioeconomic status, father’s ethnicity, and age and sex imbalance between the samples of infants, toddlers, and pre-schoolers. Among these variables, two stand out, notably differing in the study that found direct links compared with the other two that identified indirect links between the constructs when accounting for certain variables. The first variable is the father’s educational level. Aquino et al. [61] found direct links in a sample of fathers who were mostly university graduates, whereas Bocknek et al. [62] and Planalp and Braungart-Rieker [63] reported indirect links in samples of fathers with diverse education levels (i.e., no formal education, primary, secondary, university). The existence of significant direct links, particularly among highly educated fathers, could suggest an influence of paternal education level. Alternatively, the strength of these direct links may be amplified by the higher probability of highly educated fathers taking paternity leave [67]. This could enhance opportunities for fathers to exert their influence through more frequent interactions with the infant from early infancy onward, thus reinforcing links between involvement and ER. Additional research is required to validate these speculative explanations. The second identified variable is the sex imbalance in the sample. Aquino et al.‘s [61] study is the only one among the three with an imbalance, in which 58% of the children were male. The sex distribution in the remaining two studies was roughly even, with about half of the participants being male and the other half female. The presence of significant direct links may thus be indicative of a sex difference. Nevertheless, this explanation is speculative and requires a more thorough investigation.

In summary, the results of these three included studies collectively suggest that direct links between the constructs are likely and that certain variables may moderate these links (assessment time of ER and father’s ethnicity, and assessment characteristics of ER and measured aspects of the infant’s temperament), leading to significant associations. However, the heterogeneity observed across the three studies makes it challenging to draw definitive conclusions, highlighting the need for further investigation.

#### ER as a physiological-biological indicator

The two studies [68, 69] that looked at ER as a physiological measure found no direct links between variables, but significant links appeared between the quantity of father involvement and ER when certain variables were taken into account: assessment characteristics of father involvement and assessment time of ER. Isaac et al. [68] found that greater children’s physiological stress measured via hair cortisol concentration is positively associated with fathers’ reports of more frequent authoritarian parenting and, more specifically, with fathers’ reports of higher physical coercion. Links were absent when other fathering behaviors were considered (fathers’ authoritative and permissive parenting; fathers’ non-reasoning/punitive behavior), thus revealing that they appeared according to the type of father involvement considered. Richter and Lickenbrock [69] found links between fathers’ self-reported higher involvement in caregiving (as opposed to play) and elevated average scores in infants’ cardiac regulation, specifically respiratory sinus arrhythmia, during interactions with both mothers and fathers. This finding suggests that the links were influenced by the quantity of father involvement, indicating that children mobilized greater physiological resources to regulate themselves in the presence of both parents when fathers were more involved. In the same study, correlations between father involvement (in play and care) and infant’s cardiac regulation were shown to vary in significance under certain conditions (i.e., time of measurement of the respiratory sinus arrhythmia), such that when fathers were more involved in play, infant’s cardiac regulation increased at 4 months (and not at 8 months); when fathers were more involved in care, infant’s cardiac regulation increased at 8 months (and not at 4 months), thus revealing that links appeared according to the time of the assessment of ER.

In summary, the results of these two studies suggest that direct links are unlikely and that considering certain variables may increase understanding of the occurrence of significant links between the two constructs. In light of these findings, we suggest that future studies further investigate the links between the quantity of father involvement and the physiological regulation of ER and should take into account the impact of the assessment characteristics of father involvement and the assessment time of ER.

### Quality of father involvement

#### ER as informant reports

Of the two studies that investigated informant-reported ER, one found no links [70] and one found no direct significant links between variables, but significant links appeared between the greater quality of father involvement and better child ER when the assessed aspects of father involvement and ER were considered [71]. Specifically, Altenburger and Schoppe-Sullivan [71] found a positive link between fathers’ reports of children’s regulatory capacity and only one of the three dimensions of father involvement measured during the interaction with the infant (positive affect, and not sensitivity and detachment). When comparing the two studies, we observed a notable degree of heterogeneity, underscoring the need for caution in formulating definitive conclusions. Specifically, as outlined by Olofson and Schoppe-Sullivan [70] and Altenburger and Schoppe-Sullivan [71], the two studies exhibit significant differences in terms of the age category of the sampled children (infants vs. toddlers), sample size (*n* = 182 vs. *n* = 62), timing of ER assessment (3 months vs. 12–18 months), and father involvement assessment (9 months vs. 12–18 months), as well as the designated informant for ER (father vs. mother). These variations and their potential impact on the study outcomes warrant thorough investigation by future research endeavors to elucidate the findings of this review. Despite their heterogeneity, the results of the two included studies suggest that direct links are unlikely, and significant associations emerge when specific aspects of father involvement and ER are considered. Consequently, there is a pressing need for further research to explore the associations between the quality of father involvement and a child’s ER, as reported by informants.

#### ER as a naturalistic or laboratory observation

Of the two studies that investigated observed ER, only one found direct links, notably between the greater quality of father involvement and better child ER [61]. Aquino et al. [61] found that an increase in the quality of paternal behaviors during interaction with the child increased the child’s ability to regulate with the father. In the other study, Lunkenheimer et al. [72] found that higher quality of paternal behaviors during interaction with the infant positively influences the infant’s ER, but only when the infant regulates during interaction with the mother. These findings underscore the need to consider the child’s ER when interacting with each parent, as suggested by previous studies (for a review, see [73]). This approach will help identify direct links between the father and child, as well as shed light on indirect influences that may be related to maternal variables.

In comparing the two studies, we observed notable differences in two variables between them. These two variables may have acted as a moderator on the links found in each study, and we suggest further research to consider their influence on the investigated links. The first variable was the assessment time of both constructs. Aquino et al. [61] found direct links when assessing the two constructs when the child was between 8 and 24 months old, whereas Lunkenheimer et al. [72] conducted assessments at 36 months. This suggests that a direct link may be more likely in children who are less than 2 years old. The second variable was the characteristics of the ER assessment. Aquino et al. [61] found direct links in a study that observed ER during a frustrating task and in the absence of both parents. Conversely, in Lunkenheimer et al.‘s [72] study, in which the child interacted with both parents in a non-stressful situation, no direct links were found. We speculate that in more arousing situations (e.g., those that are stressful), the child may activate regulatory processes learned during shared moments with the father, making links more likely to be significant. This explanation, requiring further investigation, is inspired by theories suggesting that fathers contribute to more challenging and stimulating interactions (social role theory [74]) and engage in more physically stimulating and unpredictable play (theory of the father-child activation relationship [26]).

In summary, despite definitive conclusions being limited by the heterogeneity of the two included studies, their results suggest the likelihood of direct links and indicate that certain variables, such as the person with whom the child interacts, may moderate these links, leading to significant associations.

#### ER as a physiological-biological indicator

The only study that investigated the physiological regulation of emotions found significant links between the greater quality of father involvement and poorer ER (higher total cortisol output [75]). The results of this particular study make it challenging to draw any conclusions. We encourage caution in interpreting them, as the physiological indicator of ER (cortisol) was measured during interactions with mothers, limiting our understanding of how father involvement directly influences children’s ER. In this study, the negative links found might reflect more of the influence of maternal variables than paternal ones, such as the impact of maternal behavior on children’s physiological ER during interactions with their mothers. Given these considerations, we recommend further research to provide deeper insights into the links between the quality of father involvement and a child’s physiological ER. Moreover, these future investigations should assess physiological indicators of ER when the child interacts with the father.

## Discussion

The results of this review on the associations between father involvement and ER in early childhood are heterogeneous. However, some general conclusions may be drawn about these associations and the measurement methods used to assess them. We discuss below the available information about the results by referring to the category of father involvement (i.e., quantity and quality) and ER (i.e., as informant reports, naturalistic or laboratory observation, and physiological-biological indicators).

Overall, the studies we have reviewed indicate that the quantity and quality of father involvement are not directly associated with a child’s ER during early childhood. Indeed, only one study reported direct associations between the quantity and quality of father involvement, on the one hand, and the child’s ER, on the other hand. Different explanations can be put forward to explain this lack of direct associations between the variables. A first reason could be that, although father involvement may be adequate, it may not be equivalent to the level of maternal involvement during early childhood. Despite societal and cultural shifts promoting increased father involvement, traditional family roles, in which fathers work full time and mothers serve as the primary caregivers, remain prevalent during early childhood [76]. This arrangement can affect the quantity and quality of father-child interactions, which, though adequate, may not be as influencing as mother-child interactions in reinforcing direct associations between parental involvement and ER [77, 78]. Future studies should consider investigating nontraditional family configurations, which were notably absent in this review. This exploration could help ascertain whether the strength of associations between the two constructs increases when fathers assume the primary caregiver role. For instance, a comparison between fathers who take on the primary caregiver role and work part-time or not at all could offer deeper insights into the results of this review, although recruiting such fathers may pose challenges.

Although this review showed weak direct associations between the target variables, many included studies reported significant indirect associations between greater involvement and better ER in children, that is, when other variables were taken into account (for detailed information and consideration about how these variables moderate the investigated associations, see Section: Narrative synthesis of the results). These variables, which may relate to the assessment of father involvement and ER (e.g., time, what was measured), as well as the characteristics of both the father and the child (e.g., sex and ethnicity), seem to moderate the associations between these two constructs. Further exploration of these moderating variables could offer deeper insights into their influence on the associations between father involvement and ER during early childhood.

Regarding measurement methods, this review confirms that previous research exhibits a certain heterogeneity in the assessment of father involvement and the child’s ER, notably during early childhood. Concerning the associations between these two constructs, this review suggests that the outcomes of the included studies vary based on the methodology, as the presence or absence of links between them appears to depend on the chosen measurement methods. For instance, the use of observational measures of ER seems to increase the likelihood of finding direct links with the quantity and quality of father involvement. This result underscores the importance of careful consideration in selecting methodologies and taking into account their potential impact on the investigated links. Taken together, the results of the included studies suggest certain trends in the methods used to study the associations between father involvement and ER in children’s first 5 years of life. Building on these trends, we propose considerations that merit further investigation. First, most of the results of this review relate to studies that investigated the associations between either the quantity or the quality of father involvement and the observed child’s ER during interactions, notably in interactions in which the mother is absent. This finding seems to highlight that contemporary fathers may be less difficult to recruit than those of the past, as they are more available to report their involvement with the child and to take part in research involving the extended periods required to record direct interactions with children [79]. Moreover, the increasing prevalence of observational measures in more recent research seems to reflect scholars’ effort to observe fathers’ influence on their children’s ER during interactions, despite the advantages derived from the use of questionnaires that are convenient and easy to administer and analyze [80]. Second, physiological measures of ER were uncommon in the included studies that focused on investigating the associations with the quantity of father involvement, and even less common in studies that investigated the quality of father involvement. When physiological measures were used to investigate associations with the quality of involvement, it appeared that the child tends to have a greater need to physiologically regulate emotions during interactions with a more involved father. However, this greater need to regulate emotions was found during interaction with the mother and never assessed with the father. Of the three studies utilizing physiological measures of ER, two were cross-sectional (out of the 10 included studies, only three were cross-sectional), and both assessed children’s physiological regulation only in stressful situations [see 68, 75]. In light of this information, we suggest two directions for future research. First, longitudinal studies generally provide greater insights into causal relationships than cross-sectional studies; therefore, future studies should investigate longitudinally the links between father involvement and children’s physiological regulation to provide more insights into their causal relationship over time. Second, considering that fathers and children may interact in more positive situations than stressful ones, such as during play, future studies should assess physiological regulation across a broader range of interactions to provide a more comprehensive understanding of the links between father involvement and children’s physiological regulation beyond stressful situations. Taken together, these findings about physiological measures of emotions indicate that future research should continue to investigate how both the quantity and the quality of father involvement seem to be associated with the need for children to physiologically regulate their emotions during interactions and that longitudinal studies are needed to increase the understanding of the causal relationship between the target variables. Furthermore, when placed in perspective with previous research on mother-child interactions, the findings of this review about physiological regulation may suggest a future direction for research. Although previous studies on mother-child interactions have often demonstrated associations between higher interaction quality and better physiological regulation of emotions in children [81], the results of this review seem to suggest that higher quality paternal behaviors reduce the child’s ability to physiologically regulate emotions with the mother. To fully understand whether a child’s physiological responses adapt differently depending on the involved parent and the paternal variables considered, future research must further explore patterns of child physiological regulation during interactions with each parent. Third, in the majority of the included studies, both parents provided reports on their child’s emotional regulation, with fathers serving as the primary informants regarding their involvement with the child. These included studies consistently revealed that increased father involvement generally did not show associations with the child’s ER. However, in one study, the associations appeared when considering both mother and father reports of father involvement [61]. This finding suggests that the utility of questionnaires might be limited by individual biases and that the likelihood of associations notably increases when using both parents’ reports of father involvement. One factor that might have influenced the lack of associations is how the questionnaires assessing father involvement, and ER combined various aspects into overall scores. Indeed, the results of the included studies show that previous research has used global indices of involvement and ER (perhaps in an attempt to capture all the different aspects of both constructs) and generally found no associations between them. Given that both constructs have multiple dimensions, it is plausible, although further research is needed, that aggregating diverse dimensions into a single score might constrain the understanding of each dimension and their interconnections. Future research should clarify these considerations by investigating which of these aspects of father involvement and ER (aggregated together to produce general indices) are the most informative for the associations between the constructs of interest. Finally, among the included studies, few investigated the association between the constructs of interest by using two or more measures to assess their multiple dimensions, instead focusing, for example, on the association between one dimension of father involvement and one dimension of ER (as categorized for both of them in this review). Future research should make greater use of multiple types of measures to assess the multidimension of both constructs of interest and thus fill the gaps in previous research.

This literature review entails a few limitations. First, cultural representativity is almost nonexistent due to the quasi-majority of studies having been conducted in the United States. Moreover, it is important to note that only one of the included studies used both parents to report father involvement. Future research should include measures of both parents from other countries to control possible bias that is specifically due to the mother, father, or cultural context. The categories used to synthesize the results, although useful for the aims of this review, could underrepresent the multidimensionality of both father involvement and ER, and may therefore be non-exhaustive. A certain heterogeneity in the theoretical framework in the included articles, the methodology, and the approach used to investigate the constructs of interest, as well as the absence of nontraditional family configurations, might limit the generalizability of the results and the possibility to draw clear conclusions.

## Conclusions

This literature review synthesizes existing findings in the literature about the associations between father involvement and ER during early childhood, as well as the measurement methods used to assess them. The information available in this review underscores what has been done and highlights what warrants further investigation (e.g., associations within nontraditional family configurations). In particular, this review points out that the included studies on the quantity and quality of the father’s involvement reported no direct influence on the child’s ER during early childhood. However, the positive influence of greater father involvement on ER during early childhood appeared when studies considered variables related to the assessment of father involvement and the child’s ER (e.g., time, what was measured) and to fathers and children (e.g., sex and ethnicity). Future investigations should include these variables and clarify their moderating influence on the association between the father’s involvement and the child’s socioemotional developmental outcomes. The findings of this review are mostly based on included studies that used observational measures of ER. More measures of involvement and ER reported by both parents are needed to provide insight and reduce possible bias due to the reported measures of only one parent. This review emphasizes the scarce use of physiological measures in studying children’s ER, especially in research examining how the quality of father involvement relates to it. Indeed, only one study delved into the influence of the quality of involvement in the physiological regulation of emotion. This particular study did not involve father-child interactions. Hence, we recommend that future research encompass these interactions along with mother-child interactions. This broader approach would offer a more comprehensive understanding of how a father’s involvement affects a child’s ER by taking into account potential influences between family members and considering measures related to both parents. Several studies included in this review provided little information about the characteristics of the samples; some important information, such as infants’ age, fathers’ race/ethnicity, fathers’ education level, fathers’ socioeconomic status (SES), fathers’ age, parents’ living arrangements, fathers’ marital status, and parents’ sexual orientation, were not consistently reported across the included articles. We suggest that future studies include this information about the sample to enhance its description and enable more meaningful comparisons across studies, ultimately improving the understanding of the links between father involvement and children’s ER during early childhood. Considering that the majority of the included studies were conducted within the last two decades and primarily relied on observational measures for both constructs, this review highlights a burgeoning trend of contemporary fathers actively engaging in research related to early childhood fatherhood. This trend may reflect fathers’ growing interest in understanding their role during this crucial developmental stage and, more generally, their awareness regarding the influence of their involvement on children’s socioemotional development.

## Electronic supplementary material

Below is the link to the electronic supplementary material.

Supplementary Material 1

Supplementary Material 2

## Data Availability

Data is provided within the manuscript or supplementary information files.

## Abbreviations

ER: Emotion regulation
