# Supplementary material for: Father involvement and emotion regulation during early childhood: a systematic review
Source: BMC Psychol. 2024 Nov 19;12:675. doi: 10.1186/s40359-024-02182-x (PMC11575111; doi:10.1186/s40359-024-02182-x)
Supplement: Supplementary file 1 — Supplementary Material 1 [file 40359_2024_2182_MOESM1_ESM.docx]

# Supplementary material

## Search Algorithms

### PubMed. (((((("Fathers"[Mesh]) OR "Paternal Behavior"[Mesh])) OR (Cohabitation[Title/Abstract] OR "Father* Accessibility"[Title/Abstract] OR "Father* Engagement"[Title/Abstract] OR "Father* Involvement"[Title/Abstract] OR "Father* responsibility"[Title/Abstract] OR Fathers[Title/Abstract] OR Involvement[Title/Abstract] OR "Parental Investment"[Title/Abstract] OR Partner[Title/Abstract] OR "Partner* Accessibility"[Title/Abstract] OR "Partner* Engagement"[Title/Abstract] OR "Partner* Involvement"[Title/Abstract] OR "Partner* Responsibility"[Title/Abstract] OR "Patern* Accessibility"[Title/Abstract] OR "Patern* Engagement"[Title/Abstract] OR "Patern* Involvement"[Title/Abstract] OR "Patern* Responsibility"[Title/Abstract] OR "Paternal Behavior"[Title/Abstract]))) AND ((((((((("Child Care"[Mesh]) OR "Child Rearing"[Mesh]) OR "Father-Child Relations"[Mesh]) OR "Infant Care"[Mesh]) OR "Parent-Child Relations"[Mesh]) OR "Parenting"[Mesh]) OR "Paternal Behavior"[Mesh])) OR ("Bab* Care*"[Title/Abstract] OR "Bab* Day* Care*"[Title/Abstract] OR "Care behavior"[Title/Abstract] OR "Caring Behav*"[Title/Abstract] OR "Child Care"[Title/Abstract] OR "Child Day Care"[Title/Abstract] OR "Child rearing"[Title/Abstract] OR "Child* Care*"[Title/Abstract] OR "Child* Day* Care*"[Title/Abstract] OR Childrearin*[Title/Abstract] OR "Childrearing Attitudes"[Title/Abstract] OR "Childrearing Practices"[Title/Abstract] OR "Father Child Communication"[Title/Abstract] OR "Father Child Relations"[Title/Abstract] OR "Father* Bab*"[Title/Abstract] OR "Father* Bab* Relation*"[Title/Abstract] OR "Father* Child*"[Title/Abstract] OR "Father* Child* Relation*"[Title/Abstract] OR "Father* Infant*"[Title/Abstract] OR "Father* Infant* Relation*"[Title/Abstract] OR "Father*-Bab*"[Title/Abstract] OR "Father*-Child*"[Title/Abstract] OR "Father*-Infant*"[Title/Abstract] OR Fathering[Title/Abstract] OR "Infant Care"[Title/Abstract] OR "Infant* Care*"[Title/Abstract] OR "Infant* Day* Care*"[Title/Abstract] OR "Parent Child Communication"[Title/Abstract] OR "Parent Child Relations"[Title/Abstract] OR "Parent* Bab*"[Title/Abstract] OR "Parent* Child*"[Title/Abstract] OR "Parent* Infant*"[Title/Abstract] OR "Parent*-Bab*"[Title/Abstract] OR "Parent*-Child*"[Title/Abstract] OR "Parent*-Infant*"[Title/Abstract] OR Parenting[Title/Abstract] OR "Partner* Bab*"[Title/Abstract] OR "Partner* Child*"[Title/Abstract] OR "Partner* Infant*"[Title/Abstract] OR "Partner*-Bab*"[Title/Abstract] OR "Partner*-Child*"[Title/Abstract] OR "Partner*-Infant*"[Title/Abstract] OR "Paternal behavior"[Title/Abstract] OR "Social[Title/Abstract] AND Interpersonal Measures"[Title/Abstract]))) AND (((((((((("Autonomic Nervous System"[Mesh]) OR "Electrocardiography"[Mesh]) OR "Emotional Adjustment"[Mesh]) OR "Emotional Regulation"[Mesh]) OR "Hypothalamo-Hypophyseal System"[Mesh]) OR "Parasympathetic Nervous System"[Mesh]) OR "Psychophysiology"[Mesh]) OR "Vagus Nerve"[Mesh])) OR ("Affect Regulation"[Title/Abstract] OR ANS[Title/Abstract] OR "Autonomic Nervous System"[Title/Abstract] OR "Cardiovascular reactivity"[Title/Abstract] OR "Cardiovascular response"[Title/Abstract] OR "Cortisol reactivity"[Title/Abstract] OR "Early Emotion* Experience*"[Title/Abstract] OR "Early Regulatory"[Title/Abstract] OR "Emotional Development"[Title/Abstract] OR ECG[Title/Abstract] OR EKG[Title/Abstract] OR "Electrocardio*"[Title/Abstract] OR Electrocardiogram[Title/Abstract] OR Electrocardiography[Title/Abstract] OR "Emotion regulation"[Title/Abstract] OR "Emotion* adjustment"[Title/Abstract] OR "Emotional Adaptation"[Title/Abstract] OR "Emotional Adjustment"[Title/Abstract] OR "Emotional Control"[Title/Abstract] OR "Emotional Development"[Title/Abstract] OR "Emotional Regulation"[Title/Abstract] OR "Heart Rate Variability"[Title/Abstract] OR "HPA axis"[Title/Abstract] OR "HTPA axis"[Title/Abstract] OR "Hypothalamo-Hypophyseal System"[Title/Abstract] OR "Hypothalamus hypophysis system"[Title/Abstract] OR "Parasympathetic Nervous System"[Title/Abstract] OR Psychophysiology[Title/Abstract] OR "Regulat* emotion*"[Title/Abstract] OR RMSSD[Title/Abstract] OR RSA[Title/Abstract] OR "Self-Regulation"[Title/Abstract] OR "Socioemotional Development"[Title/Abstract] OR "Socioemotional Functioning"[Title/Abstract] OR "Vagal Functioning"[Title/Abstract] OR "Vagal Regulation"[Title/Abstract] OR "Vagal Suppression"[Title/Abstract] OR "Vagal tone regulation"[Title/Abstract] OR "Vagal Withdrawal"[Title/Abstract] OR "Vagus Nerve"[Title/Abstract] OR "Vagus Tone"[Title/Abstract]))

### PsycInfo. (((Index Terms: ("Autonomic Nervous System") OR Index Terms: ("Cardiovascular reactivity") OR Index Terms: ("Electrocardiography") OR Index Terms: ("Emotional Control") OR Index Terms: ("Emotional Development") OR Index Terms: ("Emotional Regulation") OR Index Terms: ("Heart Rate Variability") OR Index Terms: ("Parasympathetic Nervous System") OR Index Terms: (Psychophysiology) OR Index Terms: ("Socioemotional Functioning") OR Index Terms: ("Vagus Nerve")) OR (abstract: ("Affect Regulation") OR abstract: (ANS) OR abstract: ("Autonomic Nervous System") OR abstract: ("Cardiovascular reactivity") OR abstract: ("Cardiovascular response") OR abstract: ("Cortisol reactivity") OR abstract: ("Early Emotion* Experience*") OR abstract: ("Early Regulatory") OR abstract: ("Emotional Development") OR abstract: (ECG) OR abstract: (EKG) OR abstract: ("Electrocardio*") OR abstract: (Electrocardiogram) OR abstract: (Electrocardiography) OR abstract: ("Emotion regulation") OR abstract: ("Emotion* adjustment") OR abstract: ("Emotional Adaptation") OR abstract: ("Emotional Adjustment") OR abstract: ("Emotional Control") OR abstract: ("Emotional Development") OR abstract: ("Emotional Regulation") OR abstract: ("Heart Rate Variability") OR abstract: ("HPA axis") OR abstract: ("HTPA axis") OR abstract: ("Hypothalamo-Hypophyseal System") OR abstract: ("Hypothalamus hypophysis system") OR abstract: ("Parasympathetic Nervous System") OR abstract: (Psychophysiology) OR abstract: ("Regulat* emotion*") OR abstract: (RMSSD) OR abstract: (RSA) OR abstract: ("Self-Regulation") OR abstract: ("Socioemotional Development") OR abstract: ("Socioemotional Functioning") OR abstract: ("Vagal Functioning") OR abstract: ("Vagal Regulation") OR abstract: ("Vagal Suppression") OR abstract: ("Vagal tone regulation") OR abstract: ("Vagal Withdrawal") OR abstract: ("Vagus Nerve") OR abstract: ("Vagus Tone")) OR (title: ("Affect Regulation") OR title: (ANS) OR title: ("Autonomic Nervous System") OR title: ("Cardiovascular reactivity") OR title: ("Cardiovascular response") OR title: ("Cortisol reactivity") OR title: ("Early Emotion* Experience*") OR title: ("Early Regulatory") OR title: ("Emotional Development") OR title: (ECG) OR title: (EKG) OR title: ("Electrocardio*") OR title: (Electrocardiogram) OR title: (Electrocardiography) OR title: ("Emotion regulation") OR title: ("Emotion* adjustment") OR title: ("Emotional Adaptation") OR title: ("Emotional Adjustment") OR title: ("Emotional Control") OR title: ("Emotional Development") OR title: ("Emotional Regulation") OR title: ("Heart Rate Variability") OR title: ("HPA axis") OR title: ("HTPA axis") OR title: ("Hypothalamo-Hypophyseal System") OR title: ("Hypothalamus hypophysis system") OR title: ("Parasympathetic Nervous System") OR title: (Psychophysiology) OR title: ("Regulat* emotion*") OR title: (RMSSD) OR title: (RSA) OR title: ("Self-Regulation") OR title: ("Socioemotional Development") OR title: ("Socioemotional Functioning") OR title: ("Vagal Functioning") OR title: ("Vagal Regulation") OR title: ("Vagal Suppression") OR title: ("Vagal tone regulation") OR title: ("Vagal Withdrawal") OR title: ("Vagus Nerve") OR title: ("Vagus Tone"))) AND (PublicationTypeFilt: "Peer Reviewed Journal")) AND (((Index Terms: ("Child Care") OR Index Terms: ("Child Day Care") OR Index Terms: ("Child rearing") OR Index Terms: ("Childrearing Attitudes") OR Index Terms: ("Childrearing Practices") OR Index Terms: ("Father Child Communication") OR Index Terms: ("Father Child Relations") OR Index Terms: ("Infant care") OR Index Terms: ("Parent Child Communication") OR Index Terms: ("Parent Child Relations") OR Index Terms: (Parenting) OR Index Terms: ("Social and Interpersonal Measures")) OR (abstract: ("Bab* Care*") OR abstract: ("Bab* Day* Care*") OR abstract: ("Care behavior") OR abstract: ("Caring Behav*") OR abstract: ("Child Care") OR abstract: ("Child Day Care") OR abstract: ("Child rearing") OR abstract: ("Child* Care*") OR abstract: ("Child* Day* Care*") OR abstract: (Childrearin*) OR abstract: ("Childrearing Attitudes") OR abstract: ("Childrearing Practices") OR abstract: ("Father Child Communication") OR abstract: ("Father Child Relations") OR abstract: ("Father* Bab*") OR abstract: ("Father* Bab* Relation*") OR abstract: ("Father* Child*") OR abstract: ("Father* Child* Relation*") OR abstract: ("Father* Infant*") OR abstract: ("Father* Infant* Relation*") OR abstract: ("Father*-Bab*") OR abstract: ("Father*-Child*") OR abstract: ("Father*-Infant*") OR abstract: (Fathering) OR abstract: ("Infant Care") OR abstract: ("Infant* Care*") OR abstract: ("Infant* Day* Care*") OR abstract: ("Parent Child Communication") OR abstract: ("Parent Child Relations") OR abstract: ("Parent* Bab*") OR abstract: ("Parent* Child*") OR abstract: ("Parent* Infant*") OR abstract: ("Parent*-Bab*") OR abstract: ("Parent*-Child*") OR abstract: ("Parent*-Infant*") OR abstract: (Parenting) OR abstract: ("Partner* Bab*") OR abstract: ("Partner* Child*") OR abstract: ("Partner* Infant*") OR abstract: ("Partner*-Bab*") OR abstract: ("Partner*-Child*") OR abstract: ("Partner*-Infant*") OR abstract: ("Paternal behavior") OR abstract: ("Social and Interpersonal Measures")) OR (title: ("Bab* Care*") OR title: ("Bab* Day* Care*") OR title: ("Care behavior") OR title: ("Caring Behav*") OR title: ("Child Care") OR title: ("Child Day Care") OR title: ("Child rearing") OR title: ("Child* Care*") OR title: ("Child* Day* Care*") OR title: (Childrearin*) OR title: ("Childrearing Attitudes") OR title: ("Childrearing Practices") OR title: ("Father Child Communication") OR title: ("Father Child Relations") OR title: ("Father* Bab*") OR title: ("Father* Bab* Relation*") OR title: ("Father* Child*") OR title: ("Father* Child* Relation*") OR title: ("Father* Infant*") OR title: ("Father* Infant* Relation*") OR title: ("Father*-Bab*") OR title: ("Father*-Child*") OR title: ("Father*-Infant*") OR title: (Fathering) OR title: ("Infant Care") OR title: ("Infant* Care*") OR title: ("Infant* Day* Care*") OR title: ("Parent Child Communication") OR title: ("Parent Child Relations") OR title: ("Parent* Bab*") OR title: ("Parent* Child*") OR title: ("Parent* Infant*") OR title: ("Parent*-Bab*") OR title: ("Parent*-Child*") OR title: ("Parent*-Infant*") OR title: (Parenting) OR title: ("Partner* Bab*") OR title: ("Partner* Child*") OR title: ("Partner* Infant*") OR title: ("Partner*-Bab*") OR title: ("Partner*-Child*") OR title: ("Partner*-Infant*") OR title: ("Paternal behavior") OR title: ("Social and Interpersonal Measures"))) AND (PublicationTypeFilt: "Peer Reviewed Journal")) AND (((Index Terms: ("Fathers") OR Index Terms: ("Involvement") OR Index Terms: ("Parental Investment") OR Index Terms: ("Partner") OR Index Terms: ("Paternal Behavior")) OR (abstract: (Cohabitation) OR abstract: ("Father* Accessibility") OR abstract: ("Father* Engagement") OR abstract: ("Father* Involvement") OR abstract: ("Father* responsibility") OR abstract: (Fathers) OR abstract: (Involvement) OR abstract: ("Parental Investment") OR abstract: (Partner) OR abstract: ("Partner* Accessibility") OR abstract: ("Partner* Engagement") OR abstract: ("Partner* Involvement") OR abstract: ("Partner* Responsibility") OR abstract: ("Patern* Accessibility") OR abstract: ("Patern* Engagement") OR abstract: ("Patern* Involvement") OR abstract: ("Patern* Responsibility") OR abstract: ("Paternal Behavior")) OR (title: (Cohabitation) OR title: ("Father* Accessibility") OR title: ("Father* Engagement") OR title: ("Father* Involvement") OR title: ("Father* responsibility") OR title: (Fathers) OR title: (Involvement) OR title: ("Parental Investment") OR title: (Partner) OR title: ("Partner* Accessibility") OR title: ("Partner* Engagement") OR title: ("Partner* Involvement") OR title: ("Partner* Responsibility") OR title: ("Patern* Accessibility") OR title: ("Patern* Engagement") OR title: ("Patern* Involvement") OR title: ("Patern* Responsibility") OR title: ("Paternal Behavior"))) AND (PublicationTypeFilt: "Peer Reviewed Journal"))

### EMBASE. ('autonomic nervous system'/exp OR 'cardiovascular response'/exp OR 'electrocardiogram'/exp OR 'electrocardiography'/exp OR 'emotion regulation'/exp OR 'emotional control'/exp OR 'emotional development'/exp OR 'heart rate variability'/exp OR 'hypothalamus hypophysis system'/exp OR 'psychophysiology'/exp OR 'socioemotional development'/exp OR 'vagus nerve'/exp OR 'vagus tone'/exp OR 'affect regulation':ab,ti OR ans:ab,ti OR 'autonomic nervous system':ab,ti OR 'cardiovascular reactivity':ab,ti OR 'cardiovascular response':ab,ti OR 'cortisol reactivity':ab,ti OR 'early emotion* experience*':ab,ti OR 'early regulatory':ab,ti OR ecg:ab,ti OR ekg:ab,ti OR 'electrocardio*':ab,ti OR electrocardiogram:ab,ti OR electrocardiography:ab,ti OR 'emotion regulation':ab,ti OR 'emotion* adjustment':ab,ti OR 'emotional adaptation':ab,ti OR 'emotional adjustment':ab,ti OR 'emotional control':ab,ti OR 'emotional development':ab,ti OR 'emotional regulation':ab,ti OR 'heart rate variability':ab,ti OR 'hpa axis':ab,ti OR 'htpa axis':ab,ti OR 'hypothalamo-hypophyseal system':ab,ti OR 'hypothalamus hypophysis system':ab,ti OR 'parasympathetic nervous system':ab,ti OR psychophysiology:ab,ti OR 'regulat* emotion*':ab,ti OR rmssd:ab,ti OR rsa:ab,ti OR 'self-regulation':ab,ti OR 'socioemotional development':ab,ti OR 'socioemotional functioning':ab,ti OR 'vagal functioning':ab,ti OR 'vagal regulation':ab,ti OR 'vagal suppression':ab,ti OR 'vagal tone regulation':ab,ti OR 'vagal withdrawal':ab,ti OR 'vagus nerve':ab,ti OR 'vagus tone':ab,ti) AND ('care behavior'/exp OR 'child care'/exp OR 'paternal behavior'/exp OR 'child rearing'/exp OR 'father child relation'/exp OR 'infant care'/exp OR 'bab* care*':ab,ti OR 'bab* day* care*':ab,ti OR 'care behavior':ab,ti OR 'caring behav*':ab,ti OR 'child care':ab,ti OR 'child day care':ab,ti OR 'child rearing':ab,ti OR 'child* care*':ab,ti OR 'child* day* care*':ab,ti OR childrearin*:ab,ti OR 'childrearing attitudes':ab,ti OR 'childrearing practices':ab,ti OR 'father child communication':ab,ti OR 'father child relations':ab,ti OR 'father* bab*':ab,ti OR 'father* bab* relation*':ab,ti OR 'father* child*':ab,ti OR 'father* child* relation*':ab,ti OR 'father* infant*':ab,ti OR 'father* infant* relation*':ab,ti OR 'father*-bab*':ab,ti OR 'father*-child*':ab,ti OR 'father*-infant*':ab,ti OR fathering:ab,ti OR 'infant care':ab,ti OR 'infant* care*':ab,ti OR 'infant* day* care*':ab,ti OR 'parent child communication':ab,ti OR 'parent child relations':ab,ti OR 'parent* bab*':ab,ti OR 'parent* child*':ab,ti OR 'parent* infant*':ab,ti OR 'parent*-bab*':ab,ti OR 'parent*-child*':ab,ti OR 'parent*-infant*':ab,ti OR parenting:ab,ti OR 'partner* bab*':ab,ti OR 'partner* child*':ab,ti OR 'partner* infant*':ab,ti OR 'partner*-bab*':ab,ti OR 'partner*-child*':ab,ti OR 'partner*-infant*':ab,ti OR 'paternal behavior':ab,ti OR 'social and interpersonal measures':ab,ti) AND ('cohabitation'/exp OR 'father'/exp OR 'partner'/exp OR 'paternal behavior'/exp OR cohabitation:ab,ti OR 'father* accessibility':ab,ti OR 'father* engagement':ab,ti OR 'father* involvement':ab,ti OR 'father* responsibility':ab,ti OR fathers:ab,ti OR involvement:ab,ti OR 'parental investment':ab,ti OR partner:ab,ti OR 'partner* accessibility':ab,ti OR 'partner* engagement':ab,ti OR 'partner* involvement':ab,ti OR 'partner* responsibility':ab,ti OR 'patern* accessibility':ab,ti OR 'patern* engagement':ab,ti OR 'patern* involvement':ab,ti OR 'patern* responsibility':ab,ti OR 'paternal behavior':ab,ti)

### Web of science. (Cohabitation OR “Father* Accessibility” OR “Father* Engagement” OR “Father* Involvement” OR “Father* responsibility” OR Fathers OR Involvement OR “Parental Investment” OR Partner OR “Partner* Accessibility” OR “Partner* Engagement” OR “Partner* Involvement” OR “Partner* Responsibility” OR “Patern* Accessibility” OR “Patern* Engagement” OR “Patern* Involvement” OR “Patern* Responsibility” OR “Paternal Behavior”) AND (“Bab* Care*” OR “Bab* Day* Care*” OR “Care behavior” OR “Caring Behav*” OR “Child Care” OR “Child Day Care” OR “Child rearing” OR “Child* Care*” OR “Child* Day* Care*” OR Childrearin* OR “Childrearing Attitudes” OR “Childrearing Practices” OR “Father Child Communication” OR “Father Child Relations” OR “Father* Bab*” OR “Father* Bab* Relation*” OR “Father* Child*” OR “Father* Child* Relation*” OR “Father* Infant*” OR “Father* Infant* Relation*” OR “Father*-Bab*” OR “Father*-Child*” OR “Father*-Infant*” OR Fathering OR “Infant Care” OR “Infant* Care*” OR “Infant* Day* Care*” OR “Parent Child Communication” OR “Parent Child Relations” OR “Parent* Bab*” OR “Parent* Child*” OR “Parent* Infant*” OR “Parent*-Bab*” OR “Parent*-Child*” OR “Parent*-Infant*” OR Parenting OR “Partner* Bab*” OR “Partner* Child*” OR “Partner* Infant*” OR “Partner*-Bab*” OR “Partner*-Child*” OR “Partner*-Infant*” OR “Paternal behavior” OR “Social and Interpersonal Measures”) AND (“Affect Regulation” OR ANS OR “Autonomic Nervous System” OR “Cardiovascular reactivity” OR “Cardiovascular response” OR “Cortisol reactivity” OR “Early Emotion* Experience*” OR “Early Regulatory” OR “Emotional Development” OR ECG OR EKG OR “Electrocardio*” OR Electrocardiogram OR Electrocardiography OR “Emotion regulation” OR “Emotion* adjustment” OR “Emotional Adaptation” OR “Emotional Adjustment” OR “Emotional Control” OR “Emotional Development” OR “Emotional Regulation” OR “Heart Rate Variability” OR “HPA axis” OR “HTPA axis” OR “Hypothalamo-Hypophyseal System” OR “Hypothalamus hypophysis system” OR “Parasympathetic Nervous System” OR Psychophysiology OR “Regulat* emotion*” OR RMSSD OR RSA OR “Self-Regulation” OR “Socioemotional Development” OR “Socioemotional Functioning” OR “Vagal Functioning” OR “Vagal Regulation” OR “Vagal Suppression” OR “Vagal tone regulation” OR “Vagal Withdrawal” OR “Vagus Nerve” OR “Vagus Tone”)
