# Supplementary material for: Father involvement and emotion regulation during early childhood: a systematic review
Source: BMC Psychol. 2024 Nov 19;12:675. doi: 10.1186/s40359-024-02182-x (PMC11575111; doi:10.1186/s40359-024-02182-x)
Supplement: Supplementary file 2 — Supplementary Material 2 [file 40359_2024_2182_MOESM2_ESM.docx]

**A2 Detailed information from the studies**

*Detailed characteristics of the studies*

| **Authors** | **Years** | **Infants/toddlers' age** | **Fathers' age** | **Study design** | **Sample size** | **Collection period of data** |
| --- | --- | --- | --- | --- | --- | --- |
| De Stasio et al. [60] | 2020 | Child, 18–36 months (60% male) | Fathers, 34 years mean | Cross-sectional | 80 | 2016-2017 |
| Aquino et al. [61] | 2023 | Infants, 8 and 24 months (58% male) | Fathers, 31 years mean | Longitudinal | 124 | N/A |
| Bocknek et al. [62] | 2014 | Child, 14–36 months (about 50% male) | Fathers, 25.51 years mean (at child's birth) | Longitudinal | 1477 | 1996-1998 |
| Planalp & Braungart-Rieker [63] | 2015 | Child, 3–7 months (52.6% female) | Fathers, 30.79 years mean | Longitudinal | 135 | N/A |
| Isaac et al. [68] | 2023 | Child, 4 years mean (43.6% female) | Fathers, 35.86 years mean | Cross-sectional | 98 | 2016-2019 |
| Richter & Lickenbrock [69] | 2021 | Child, 4–8 months (59.3% male) | Fathers, 32.14 years mean | Longitudinal | 91 | 2018-2021 |
| Olofson and Schoppe-Sullivan [70] | 2022 | Child, 16.37 months mean (64.51% male) | Fathers, 29.40 years mean | Longitudinal | 62 | 2008-2014 |
| Altenburger & Schoppe-Sullivan [71] | 2020 | Infants, 3–9 months (52.8% male) | Fathers, 30.20 years mean | Longitudinal | 182 | 2008-2010 |
| Lunkenheimer et al. [72] | 2020 | Child, 41.40 months mean (about 50% female) | Fathers, age data not indicated | Longitudinal | 173 | From 1999 (longitudinal data) - no information was found about the end of the collection. |
| Burniston et al. [75] | 2023 | Child, 42.36 months mean (about 57% female) | Fathers, age data not indicated | Cross-sectional | 42 | 2017-2020 |

*Detailed ER information in the studies*

| **Authors** | **Type of ER measure** | **ER measure** | **Assessment time of child's ER** | **Informant(s) of EM** | **Assessment characteristics of EM** | **Coder(s) of observed EM** |
| --- | --- | --- | --- | --- | --- | --- |
| De Stasio et al. [60] | Informant-reports | The Lability/Negativity subscale and the Emotion Regulation subscale of the Emotion Regulation Checklist (ERC) | 18-36 months | Both parents | N/A | N/A |
| Aquino et al. [61] | Naturalistic or laboratory observation | Underregulation scale of the Children's Emotion regulation Scales | 24 months | N/A | Two challenging and frustrating tasks in the presence of the only researcher | Researchers |
| Bocknek et al. [62] | Naturalistic or laboratory observation | Emotion Regulation rating subscale during an assessment | 14-, 24-, 36 months birthday | N/A | No interactions with mothers | N/A |
| Planalp & Braungart-Rieker [63] | Naturalistic or laboratory observation | Infant regulatory behaviors during the Still Face Paradigm (SFP) | 3, 5 and 7 months | N/A | Still Face Paradigm (SFP), with father | Researchers |
| Isaac et al. [68] | Physiological-biological indicators | Children's physiological stress measured via hair cortisol concentration | Between 3 and 5 years | N/A | During the lab visit, there were no interactions with parents | N/A |
| Richter & Lickenbrock [69] | Physiological-biological indicators | Respiratory sinus arrhythmia (RSA) | 4-8 months | N/A | Still Face Paradigm (SFP) and 3-min recovery task | N/A |
| Olofson and Schoppe-Sullivan [70] | Informant-reports | Dysregulation score of the Infant-Toddler Social-Emotional Assessment | 12-18 months | Mother | N/A | N/A |
| Altenburger & Schoppe-Sullivan [71] | Informant-reports | Negative emotionality and orienting and regulatory capacity scores from the Revised Infant Behavior Questionnaire – Very Short Form | 3 months | Father | N/A | N/A |
| Lunkenheimer et al. [72] | Naturalistic or laboratory observation | Child negative emotion arousal with father and mothers during the dyadic block design task | 3 years | N/A | With the father and mother during the dyadic block design task. | Researchers |
| Burniston et al. [75] | Physiological-biological indicators | Children's physiological stress responses | 3-4 years | N/A | Stressing situation during a visit with the mother. No father at the time of the measure of cortisol | N/A |

*Detailed FI information in the studies*

| **Authors** | **Type of FI measure** | **FI measure** | **Type of FI measure** | **Assessment time of FI** | **Informant(s) of FI** | **Coder(s) of observed FI** | **Domains of FI** |
| --- | --- | --- | --- | --- | --- | --- | --- |
| De Stasio et al. [60] | Quantity | (1) Father involvement in every day; and (2) bedtime care from the Social Provisions Scales (SPS) | Report | 18-36 months | Father | N/A | Bedtime care routine |
| Aquino et al. [61] | Quantity and Quality | Quantity: Father's involvement in infant caregiving - Quality: (1) The Infant Caregiving Scales to assess fathers' emotionally disengaged interactions with the infant at 8 months, (2) The Parents' Responses to Children's Emotions Rating Scales to assess fathers' minimizing responses to toddlers' emotional distress at 24 months. | Quantity: report - Quality: observational | Quantity: 8 months - Quality: At 8 months, videotaped father and infant interaction during home visits. At 24 months, free play, clean up, and puzzle-type tasks with the father and the infant. | Mother and father | Researchers | Quantity: care during the week - Quality: Free play, clean up, and puzzle-type tasks, feeding, changing clothes |
| Bocknek et al. [62] | Quantity | (1) Father lives with the child (yes/no); (2) quantity of contact with the child | Report | 14-, 24-, 36 months birthday | Mother | N/A | Fathers' continuous physical presence/absence |
| Planalp & Braungart-Rieker [63] | Quantity | Diary-like checklist | Report | 3, 5 and 7 months | Father | N/A | Care, play, and teaching activities |
| Isaac et al. [68] | Quantity | Frequency of specific parenting practices through the Parenting Styles and Dimensions Questionnaire. | Report | Between 3 and 5 years | Father | N/A | Fathers' parenting style |
| Richter & Lickenbrock [69] | Quantity | What I Did with My Baby Checklist (care and play) | Report | 4-8 months | Father | N/A | Father's availability, care, and play activities |
| Olofson and Schoppe-Sullivan [70] | Quality | Father's parenting behavior during a laboratory visit | Observational | 12-18 months | N/A | Researchers | Fathers' parenting style |
| Altenburger & Schoppe-Sullivan [71] | Quality | Father's parenting quality during 5-min dyadic play using Parent-Child Coding Manual | Observational | 9 months | N/A | Researchers | Three parenting dimensions: sensitivity, detachment, and positive regard |
| Lunkenheimer et al. [72] | Quality | Father's emotional responsiveness and expressiveness to child emotion cues during the dyadic block design task | Observational | 3 years | N/A | Researchers | Fathers' parenting style |
| Burniston et al. [75] | Quality | Fathers' supportive emotion socialization (ES) usage during a book reading task | Observational | 3-4 years | N/A | Researchers | Reading book task |

ER: Emotion Regulation

FI: Father Involvement

N/A: Not available/applicable
